# A reference frame for blood volume in children and adolescents

**DOI:** 10.1186/1471-2431-6-3

**Published:** 2006-02-17

**Authors:** Ann Raes, Sarah Van Aken, Margarita Craen, Raymond Donckerwolcke, Johan Vande Walle

**Affiliations:** 1 Department of Paediatric Nephrology, University Hospital, Ghent, Belgium; 2 Department of Paediatric Nephrology, Academic Hospital Maastricht, Netherlands; 3 Department of Paediatric Endocrinology, University Hospital, Ghent, Belgium

## Abstract

**Background:**

Our primary purpose was to determine the normal range and variability of blood volume (BV) in healthy children, in order to provide reference values during childhood and adolescence. Our secondary aim was to correlate these vascular volumes to body size parameters and pubertal stages, in order to determine the best normalisation parameter.

**Methods:**

Plasma volume (PV) and red cell volume (RCV) were measured and F-cell ratio was calculated in 77 children with idiopathic nephrotic syndrome in drug-free remission (mean age, 9.8 ± 4.6 y). BV was calculated as the sum of PV and RCV. Due to the dependence of these values on age, size and sex, all data were normalised for body size parameters.

**Results:**

BV normalised for lean body mass (LBM) did not differ significantly by sex (p < 0.376) or pubertal stage (p < 0.180), in contrast to normalisation for the other anthropometric parameters. There was no significant difference between reference values for children and adults.

**Conclusion:**

LBM was the anthropometric index most closely correlated to vascular fluid volumes, independent of age, gender and pubertal stage.

## Background

Alterations in blood volume (BV) lead to haemodynamic adaptations in acute and chronic diseases such as insulin-dependent diabetes mellitus, cardiac, renal and/or hepatic failure, and nephrotic syndrome [1-4]. To evaluate changes in BV in children under different clinical conditions, it is necessary to obtain reference values. Despite the importance in daily clinical practice, only few direct measurements of blood volume have been performed in children. In the largest study performed to date in a paediatric population, 160 infants and children aged 1 hour to 14 years [5], BV was calculated from plasma volume and haematocrit using a fixed F-cell ratio of 0.91, derived from studies in adults [6]. The F-cell ratio, which determines the relative distribution of BV between the microvasculature and the large vessels, describes the relation between whole body and large vessel haematocrit. Due to the Fahraeus-Lindqvist effect, however, haematocrit differs significantly between large vessels and capillaries [7,8]. Use of a fixed F-cell-ratio of 0.91 not only disregards large inter-individual variability in adults [7,8], but presumes that the distribution of large and small vessels in children and adults is comparable, despite their significantly different body compositions. To obtain reliable BV measurements, plasma volume (PV) and red cell volume (RCV) [7,9] should be measured simultaneously, rather than basing BV on measurements of PV alone with a fixed F-cell ratio.

The International Committee for Standardisation in Hematology has recommended measuring RCV by radioactive chromium or technetium methods, and PV by the radioiodine labelled albumin method [10]. To date, the only data available in children were obtained in our centre, in patients with minimal change nephrotic syndrome in remission [11]. Although these findings and normalization for LBM were in accordance with findings in adults [7,9], our earlier study had major limitations. Aside from the limited number of patients included, the sex ratio of the prepubertal children was not equally distributed and the number of patients during pubertal stages 2–4 was too small to calculate a true reference frame.

The aim of the present study was to obtain a reference frame for BV in children and adolescents, to calculate the best parameter for normalisation, and to evaluate the need for the use of the combined measurement method (i.e. PV plus RCV). We extended findings in our earlier cohort of patients [11], particularly by increasing the number of adolescents in different pubertal stages and the percentage of females.

## Methods

### Volume measurements

Twelve hours prior to administration of 131I-human serum albumin (HSA), KI was administered orally to prevent thyroid uptake of radioactive iodine. On the morning of the volume determination, 5 ml venous blood was drawn for red blood cell (RBC) labelling with 51 Cr [10], and the left and right cephalic veins were catheterised. After at least 30 min of recumbency, 4 ml blood was drawn to serve as a blank and for determination of haematocrit. Each subject was administered a bolus injection of 131I-HSA in the laminar stream of 10 ml saline solution via the rubber extension of a T-extension set (Abbott), a procedure designed to avoid adhesion of the tracer to the catheter or connexion set. The syringe was rinsed by flushing twice with saline, and residual radioactivity in all used material was measured and subtracted from total counts in the syringe. Exactly 10 minutes later, 4 ml blood was drawn from the contralateral arm for counting of radioactivity. Subsequently, 51 Cr-RBC was administered by the same procedure. The radioactive doses (in msv) were calculated as 131I-HSA = 0.0061 × years and 51-Cr-RBC = 0.0073 × years. Haematocrit was determined in triplicate.

### Calculations

BV was calculated as RCV + PV [9], whole body haematocrit was calculated as RCV/BV, and F-cell ratio as whole-body haematocrit/large vessel haematocrit. Due to differences in body composition of males and prepubertal [7], pubertal and older females, BV was analysed separately for these subgroups and normalised relative to age, sex, and the most appropriate body size parameters, including body weight (BW), height (H), body surface area (BSA) and lean body mass (LBM). BSA was estimated from a normogram based on height (H) and body weight (BW) [12]. LBM was estimated from H and BW according to the formulae:

In males and prepubertal females (Tanner stage 1), LBM (a) = 0.407BW + 26.7H -19.2,

In post pubertal or older females (Tanner stage 5), LBM (b) = 0.252BW + 47.3H - 48.3.

In pubertal females (Tanner stages 2–4), LBM(c) = {LBM (a) + LBM (b)}/2

These formulae were derived from the empirical relation between total body water (TBW), BW and H and between TBW and LBM (in kg) [7,13]. Elimination of TBW gives LBM (in kg) as a function of BW (in kg) and H (in m). LBM can be used as the index for normalisation of body fluid volumes, independent of gender.

The study was approved by the ethical committee of University Hospital Ghent (1.5.643.98). All patients and/or their parents gave written informed consent.

### Study population

The study cohort consisted of 77 children with steroid responsive nephrotic syndrome, in whom vascular fluid volumes were measured during stable remission, and included the initial cohort of 31 children [11]. All patients had H, BW and BSA within the normal range (5–95 percentile) for their age, were in remission for at least 6 months and free of therapy for at least 3 months. Patients with syndromal disorders, comorbidity, or apparent dysmorphism were excluded, as were those who used diuretics, steroids, cyclosporin A, NSAIDs or antihypertensives. The baseline demographic and clinical data of the study group are shown in Table 1.

**Table 1:** Demography: characteristics of the study population

	Controls
N (M/F)	42/35
Age (yr)	9.8 ± 4.6
Length (cm)	133.2 ± 24.8
BW (kg)	35.5 ± 17.7
BSA (m^2^)	1.1 ± 0.4
BMI (kg/m^2^)	18.4 ± 4.2
LBM (kg)	29.91 ± 12.49
GFR (ml/min/1.73 m^2^)	125 ± 34
RPF (ml/min/1.73 m^2^	616 ± 158
FE Na (%)	1,2 ± 0,4
Plasma renin activity (MU/ml)	Median 32 (6–98)
Plasma aldosterone (Pg/ml)	Median 39 (5–95)

### Statistics

Data are expressed as mean ± standard deviation and compared using the Mann-Whitney U-test. Pearson's correlation coefficient and linear regression analysis among age, H, BW, BSA and LBM and the different vascular volume values (PV, RCV, BV) were used where indicated.

## Results

The population consisted of 42 males and 35 females; their baseline demographic and clinical characteristics are shown in Table 1. Their mean age was 9.8 ± 4.6 y, mean H was 133.2 ± 24.8 cm (P65-P25), mean BW was 35.5 ± 17.7 kg (P75-P30), mean BSA was 1.1 ± 0.4 m^2 ^(P70-P30) and mean BMI (w/h^2^) was 18.4 ± 4.2 kg/m^2^. Renal functional parameters, including glomerular filtration rate (GFR), renal plasma flow (RPF) and fractional excretion of sodium, were within the normal range [14]. All patients were followed up for at least 6 years after the study, during which time serum creatinine and blood pressure remained normal, indicating an absence of severe renal parenchymal damage. Plasma renin activity and aldosterone levels were within normal ranges [14], indicating none of the patients had experienced major renovascular disturbances. The values we obtained were somewhat higher than those reported in the literature, which may have been due to our samples having been taken at the time of blood pressure measurement, rather than upon arising.

**Figure 1:**
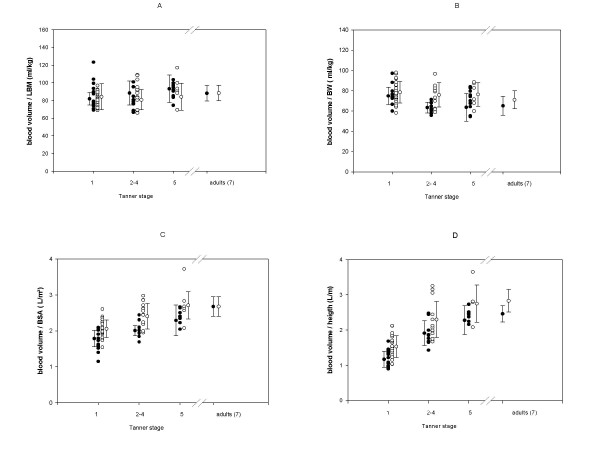
**Relationships between blood volume and different body size parameters in prepubertal (1), pubertal (stadia 2–4) and postpubertal stage (5) for girls (●) and boys (○) and adults (ref. P. Boer [7]). **A, individual data for the relationships between BV (ml) and LBM (kg). B, individual data for the relationships between BV (ml) and BW (kg). C, individual data for the relationships between BV (L) and BSA (m^2^). D, individual data for the relationships between BV (L) and height (m).

**Figure 2:**
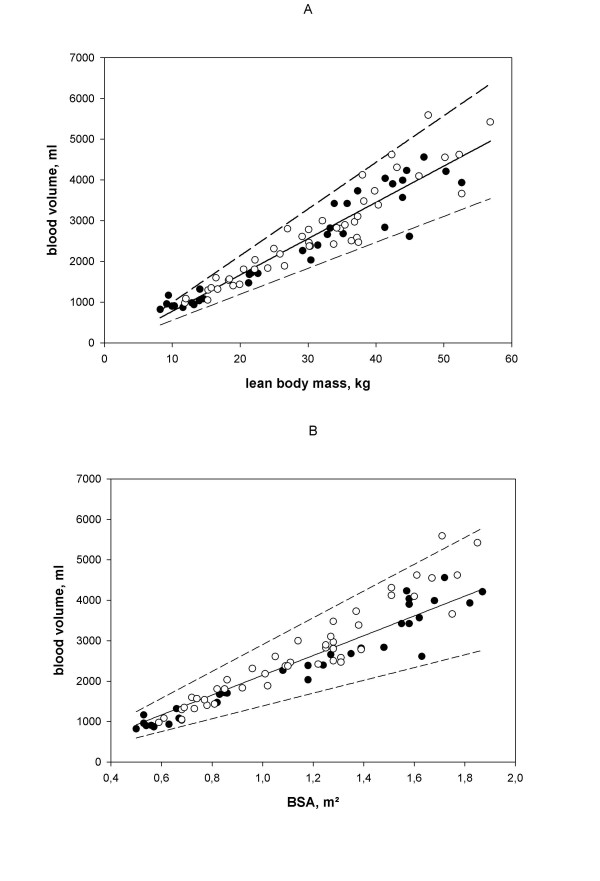
**Correlations between blood volume and lean body mass and body surface area. **A, blood volume (ml) plotted against lean body mass (kg) in girls (●) and boys (○). B, blood volume (ml) plotted against BSA (m^2^) in girls (●) and boys (○). The normal range is defined as twice the standard deviation and is identified by the dashed lines.

The mean values for PV, RCV and BV (calculated as the sum of PV and RCV), normalised for age, H, BMI, BW, BSA and LBM, are shown in Table 2, separated by sex and by pubertal stage. We observed significant differences between the sexes for body fluid volumes normalised for BMI, BW and BSA, but not when we normalised BV for H and LBM. When we assorted these values by pubertal stage, we found that only LBM show no statistical difference for gender (Fig 1a, b, c, d). The correlation coefficients for BV relative to age, BSA and LBM were of comparable magnitude and highly significant (p < 0.001), with the highest r^2 ^for BV normalised for LBM. The correlation coefficient for BV/LBM was also highly significant (r= 0.949, p < 0.001) in this paediatric population. The intercept of the regression line for BV normalised for LBM was close to 58 ml but did not pass through the origin (Table 3). Due to the absence of inherent gender-related differences, we used combined LBM data of males and females to calculate regression lines. When BSA was used, the intercepts were clearly different from zero. The regression line of BV differed significantly between the sexes (Table 3). The regression of BV on LBM and BSA is shown in Fig 2 (a,b), which also demonstrates the reference range of BV as a function of LBM and BSA. When we plotted individual data for the relationship between BV and LBM, we found that males and females were evenly distributed throughout the range of LBM (Fig 2a). The individual data for the relationship between BV and BSA are plotted in Fig 2b. We found that the F-cell ratio did not differ significantly between the sexes, being 0.83 ± 0.09 (n = 47) for males and 0.85 ± 0.08 (n = 35) for females (Table 2).

**Table 2:** Normalisation of blood volume for body size parameters. Plasma volume (PV), red cell volume (RCV) and blood volume (BV) normalized for height (L), body weight (BW), body surface area (BSA) or lean body mass (LBM) as well as the F-cell ratio of the male and female study population. Data is presented as mean and standard deviation and was analysed with a Mann-Whitney U-test

Gender		Male	Female	
N		42	35	P
PV/height (L/m)		1.30 ± 0.44	1.16 ± 0.39	NS
PV/BW (ml/kg)		52.3 ± 8.3	47.9 ± 7.7	0.011
PV/BSA (L/m^2^)		1.5 ± 0.28	1.38 ± 0.24	0.026
PV/LBM (ml/kg)		57.8 ± 9.1	58.3 ± 10.5	NS
RCV/height (L/m)		0.63 ± 0.21	0.55 ± 0.22	NS
RCV/BW (ml/kg)		25.3 ± 4.7	22.4 ± 4.88	0.002
RCV/BSA (L/m^2^)		0.74 ± 0.15	0.65 ± 0.17	0.01
RCV/LBM (ml/kg)		27.9 ± 4.3	26.9 ± 4.9	NS
BV/height (L/m)	All	1.93 ± 0.62	1.68 ± 0.58	NS
	1	1.53 ± 0.31	1.17 ± 0.23	0.001
	2–4	2.30 ± 0.51	1.91 ± 0.35	NS
	5	2.75 ± 0.53	2.28 ± 0.41	NS
		0.000^a^	0.000^a^	
	Ref. Boer (7)	2.83 ± 0.32	2.46 ± 0.23	
BV/BMI (ml/kg/m^2^)	All	143 ± 47.6	121 ± 47.2	0.035
	1	119 ± 37.5	81.9 ± 25.9	0.001
	2–4	164 ± 46.3	131 ± 14.3	NS
	5	196 ± 13.4	173 ± 36.2	NS
		0.000^a^	0.000^a^	
BV/BW (ml/kg)	All	77.4 ± 11.0	68.7 ± 11.0	0.001
	1	78.5 ± 10.6	74.9 ± 8.5	NS
	2–4	75.9 ± 12.0	63.4 ± 5.2	0.008
	5	76.4 ± 11.6	63.7 ± 13.7	NS
		NS^a^	0.007^a^	
	7	71.1 ± 8.8	65.2 ± 9.4	
BV/BSA (L/m^2^)	All	2.254 ± 0.378	1.989 ± 0.351	0.004
	1	2.059 ± 0.244	1.789 ± 0.231	0.002
	2–4	2.409 ± 0.353	2.007 ± 0.145	0.016
	5	2.711 ± 0.381	2.292 ± 0.429	0.045
		0.001^a^	0.003^a^	
	7	2.68 ± 0.27	2.40 ± 0.28	
BV/LBM (ml/kg)	All	83.4 ± 13.8	84.2 ± 14.9	NS
	1	82.0 ± 7.1	84.2 ± 14.7	NS
	2–4	88.5 ± 13.5	80.9 ± 11.4	NS
	5	93.2 ± 15.6	84.2 ± 15.2	NS
		NS^a^	NS^a^	
	7	88.2 ± 8.7	88.4 ± 8.7	
F-Cell ratio		0.83 ± 0.10	0.85 ± 0.08	NS

P < 0.05: significance level for differences between male and female population

All = combined data of different pubertal stages

1: prepubertal stage (ref. Tanner)

2–4: pubertal stage (ref. Tanner)

5: postpubertal stage (ref. Tanner)

7: ref. P. Boer

^a: ^significance level (MWU) between the different pubertal stages

**Table 3:** Correlation between blood volume and body size parameters.  Linear regression for BV versus height, body weight (BW), body surface area (BSA) and lean body mass (LBM) of the study population (n = 77). (C = complete population, M = male, F= female)

	Slope	Intercept	R	P
Height	C 42.057	-3050	0.876	<0.0001
BW	M 63.11 F 62.34	312 358	0.944 0.943	<0.0001 <0.0001
BSA	M 2836.2 F 2846.9	-669.2 -715	0.938 0.935	<0.0001 <0.0001
LBM	C 89.117	58.3	0.949	<0.0001

**Figure 3:**
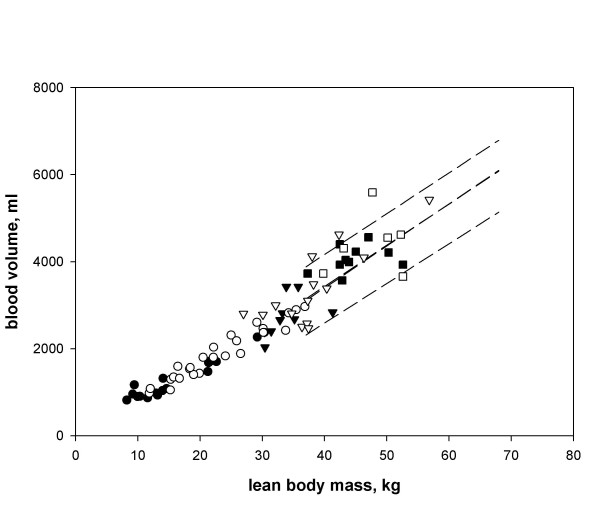
**Correlation between blood volume and lean body mass in relation to the adult references. **Blood volume (ml) plotted against LBM (kg) for the different pubertal stages for girls (Tanner stage 1: ●, 2 – 4: ▼, 5: ■) and boys (Tanner stage 1: ○, 2 – 4: ▽, 5: □). The dashed lines identify the range (twice the standard deviation) obtained in the adult population (47 M/40 F, aged 19–72 years) [7].

When we plotted the regression of BV as a function of LBM for the entire study population, in which the normal range was defined as twice the standard deviation and is identified by the dashed lines, we observed a continuum from puberty to adulthood (Fig. 3). We observed no significant difference between the regression of our data and those determined earlier in adults [7], when LBM was used. Because there was a significant difference between sexes and different pubertal stages when BV was normalized relative to BSA, with a higher BV for males, we selected LBM as the index for normalisation of body fluid volumes.

## Discussion

To our knowledge, this study is the largest to date on the direct determination of BV from PV and RCV in children and adolescents at various pubertal stages. Our pilot study in 31 patients aged 2–18 years, which measured PV and RCV, provided reference values for these parameters [11]. The aim of the earlier study was to determine whether children with idiopathic nephrotic syndrome (INS) experienced changes in blood volume, or hypovolemia, during relapses, but we found no evidence that this occurred, even during paired analysis [14]. We found, however, that LBM provided the best index for normalisation of body fluid volumes, resolving the problem of gender differences and confirming earlier findings in adults [7]. However, our earlier study had several limitations, including the small number of patients (n = 31), the predominance of males, the limited number of adolescents during pubertyand not taking into account the pubertal stages. To overcome these major limitations, we extended our study to a larger number of children (approval EC nr 1.5.643.98). Although the use of children with idiopathic nephrotic syndrome in remission as control population may be problematic when compared with "normal" children, there are ethical issues concerning studies with radiolabelled compounds in normal healthy children. This type of physiological studies is not longer defended, unless the studied children receive a direct or indirect benefit from the findings. This is also applicable to the need for a reference frame to compare values in pathological conditions.

These same ethical considerations are less applicable to nephrotic children in remission, since studies using radiolabelled compounds in these children have led to major changes in the diagnosis and treatment of hypo-/hypervolemia. Children with minimal change nephrotic syndrome in drug free remission are considered to have normal renal and haemodynamic parameters during proteinuria- and drug-free episodes [14,15]. Glomerular and tubular function are normal, as are renal histology and blood pressure, and these factors are not altered during long term follow up, suggesting that the prognosis of these patients is very good [14]. All patients in our study population were in complete remission (no proteinuria) for at least 6 months, and had received no drug therapy for at least previous 3 months. In addition, all had normal renal function, both for glomerular (GFR, RPF, FF) and tubular parameters (FE_Na_), and their plasma renin activity and aldosterone levels were within the normal range. In addition, long term follow up (> 6 y) showed no deterioration of renal function, thereby excluding severe renal parenchymal damage. This, together with the absence of clinical evidence for abnormal body composition, allows us to use this population as a reference for body fluid volumes in children. There is no evident reason why this highly selected study population cannot be considered as alternative and at least as the best reference frame available.

The aim of this study was to determine a reference frame for body fluid volumes in children and to find the best normalisation parameters for BV. In the absence of very large study groups, normalisation is unavoidable, because of the large body size and age differences in children. The use of ratios as a consequence of normalisation, however, has pitfalls [16]. In order to use a ratio successfully, the intercept of the line created by plotting one variable against another must be zero, the relationship must be a straight line and the variability around the line must be constant. This methodological problem is clearly demonstrated by comparing Figures 2A and 3. The best method for normalisation is the use of standard deviation scores (SDS) or percentiles, which are interchangeable. Because of the potential of complex interactions between sex, pubertal stage, and body composition, this would require much larger numbers of individuals, of both genders and at different pubertal states, which is not feasible in young children. Therefore, normalisation by ratios is the best available tool, despite its limitations.

We were not surprised to observe a very good linear correlation between the different vascular volumes and the parameters related to size (age, W, H, BSA, and LBM). Although correlations with other parameters (BW and BSA) were apparently limited, the larger intercept, as well as the sex and pubertal differences, make them unsuitable as simple normalisation parameters. Our finding of the highest linear correlation (R = 0.949) with LBM not only resolves the problem of sex differences, but is also in line with findings in adults [7]. When plotting individual data, however, we observed that pubertal children lay above the mean, but within the positive standard deviation area, suggesting that, during puberty, BV increases faster than LBM. Hormonal influences may be responsible for this phenomenon. In contrast, the absolute values for BV/LBM in our study population were somewhat lower, but not significantly different, than the adult values.

Despite statistical evidence in favour of the use of LBM, some critical comments must be made. Since the formula for LBM is derived from an empirical relationship, based on equations from a 20-year-old study, it is not known if this formula is still appropriate for children of this century. The equation is different for males/prepubertal females and pubertal/older females due to differences in body composition [7,13,17], but neglects the progressive changes in body composition during puberty. Because puberty is a dynamic process and body composition changes during puberty, the formula for LBM for pubertal girls should be adapted to pubertal stage. Our previous study [11] assessed a limited number of adolescents, and there was an unequal distribution between males and females, thus obscuring this finding.

Because of these serious methodological considerations regarding the use of LBM, the other normalisation parameters should be evaluated as alternatives, although it is questionable whether children of all ages can or should be "forced" to fit a single normalisation. When we normalised for BSA, BW, or H, we observed differences between males and females during pubertal stages 2–4, as well as differences with prepubertal and adult values, suggesting that none of these alternatives can be used as a single normalisation parameter. Rather, we have to take into account sex and pubertal stage differences, even if we neglect the theoretical importance of a low intercept value. This is not surprising inasmuch as the concept of LBM was created to exclude gender differences in body composition, and, as a consequence, progressive pubertal changes. This is not just a mathematical trick, but also an unavoidable methodological necessity if we want to plot males and females in one reference frame. The only alternative would be to extend the study population to create reference frames dependent on age, sex and pubertal stage. The fact that this solution is not achievable due to many practical and ethical considerations favours the use of LBM as a single parameter.

Statistical differences in the ratios of volume to various parameters of body composition may be related to differences in the derived body composition equations/nomograms rather than to true differences in RCV, PV, or BV. Significant differences in normalised values are apparently related to differences between the normalisation parameters during puberty, because BV was the same in every calculated ratio, and the best correlation was observed with derived parameters (LBM and BSA) rather than with measured variables (BW, H or age). This is not surprising, because direct measured parameters are less suitable for normalising body composition and for evaluating pathophysiological mechanisms, including the adequacy of peritoneal dialysis, perspiration, and drug distribution volume.

## Conclusion

The aim of the present study was to determine a reference frame for blood volume studies in children, using a combination of RCV and PV measurements. The wide range of F-cell ratios clearly shows that the use of plasma volume and a fixed F-cell ratio of 0.91 is not feasible. Despite its major theoretical disadvantages, the use of adapted formulas for LBM emerged as the only statistically defensible normalisation parameter, due to its high correlation factor and low intercept. LBM also has the advantage of correcting for differences between the sexes and the pubertal stages, as well as being in accordance with findings in adults. The adaptation of the LBM equation during puberty, while artificial, may resolve a methodological problem. This study offers an important tool for studying conditions in which alterations in BV may be important, including hypertension, diabetic nephropathy, capillary leak syndrome, and nephrotic and nephritic syndromes.

## Abbreviations

BSA: body surface area

LBM: lean body mass

BV: blood volume

PV: plasma volume

RCV: red cell volume

TBW: total body water

BW: body weight

P: percentile

KI: potassiumiodide

## Competing interests

The author(s) declare that they have no competing interests.

## Authors' contributions

AR contributed significantly in the design of the project, including formulation of questions and methods and design of the individual experiments, in patient recruitment, in performing the tests and experiments, in collecting the data, in the statistical analysis, in interpretation and discussion of the results and in writing the manuscript. SVA participated in the coordination of the study. MC participated in patient recruitment and in discussion of the results. RD contributed to collaboration in writing the manuscript, statistical analysis and correction of the manuscript. JVDW contributed significantly in the design of the project and its coordination, in statistical analysis, in presentation and interpretation and discussion of the results and in correction of the manuscript.

## Pre-publication history

The pre-publication history for this paper can be accessed here:


